# PA-MSHA improves prognosis of patients undergoing radical cystectomy: a retrospective cohort study using inverse probability of treatment weighting

**DOI:** 10.3389/fimmu.2024.1403302

**Published:** 2024-06-25

**Authors:** Xiaohua Zhang, Zixu Pei, Jinglei Ren, Jing Shi, Wenjun Lu, Yuan Shui, Wentao Ma, Luyang Zhang, Hui Ding, Yunxin Zhang, Junqiang Tian, Zhiping Wang

**Affiliations:** ^1^ Institute of Urology, Gansu Province Clinical Research Center for Urinary System Disease, The Second Hospital & Clinical Medical School, Lanzhou University, Lanzhou, China; ^2^ The Second Clinical Medical College of Lanzhou University, Lanzhou, Gansu, China; ^3^ The First Clinical Medical College of Lanzhou University, Lanzhou, Gansu, China

**Keywords:** Pseudomonas aeruginosa-mannose-sensitive hemagglutinin, bladder cancer, radical cystectomy, lymphatic leakage, inverse probability of treatment weighting, retrospective cohort study

## Abstract

**Objective:**

To observe the effect of Pseudomonas aeruginosa mannose-sensitive hemagglutinin (PA-MSHA) on the prognosis and the incidence of lymphatic leakage in patients undergoing radical cystectomy (RC).

**Method:**

A total of 129 patients who underwent RC in Lanzhou University Second Hospital from 2013 to 2022 were enrolled in this study. They were divided into 43 patients treated with PA-MSHA and 86 patients in the control group. Inverse probability of treatment weighting (IPTW) was applied to reduce potential selection bias. Kaplan-Meier method and Cox regression analysis were used to analyze the effect of PA-MSHA on the survival of patients and the incidence of postoperative lymphatic leakage.

**Results:**

The PA-MSHA group exhibited improved overall survival (OS) and cancer-specific survival (CSS) rates compared to the control group. The 3-year and 5-year overall survival (OS) rates for the PA-MSHA group were 69.1% and 53.2%, respectively, compared to 55.6% and 45.3% for the control group (Log-rank=3.218, P=0.072). The 3-year and 5-year cancer-specific survival (CSS) rates for the PA-MSHA group were 73.3% and 56.5%, respectively, compared to 58.0% and 47.3% for the control group (Log-rank=3.218, P=0.072). Additionally, the 3-year and 5-year progression-free survival (PFS) rates for the PA-MSHA group were 74.4% and 56.8%, respectively, compared to 57.1% and 52.2% for the control group (Log-rank=2.016, P=0.156). Multivariate Cox regression analysis indicates that lymph node metastasis and distant metastasis are poor prognostic factors for patients, while the use of PA-MSHA can improve patients’ OS (HR: 0.547, 95%CI: 0.304–0.983, P=0.044), PFS (HR: 0.469, 95%CI: 0.229–0.959, P=0.038) and CSS (HR: 0.484, 95%CI: 0.257–0.908, P=0.024). The same trend was observed in the cohort After IPTW adjustment. Although there was no significant difference in the incidence of postoperative lymphatic leakage [18.6% (8/35) vs. 15.1% (84.9%), P=0.613] and pelvic drainage volume [470 (440) ml vs. 462.5 (430) ml, P=0.814] between PA-MSHA group and control group, PA-MSHA could shorten the median retention time of drainage tube (7.0 d vs 9.0 d) (P=0.021).

**Conclusion:**

PA-MSHA may improve radical cystectomy in patients with OS, PFS, and CSS, shorten the pelvic drainage tube retention time.

## 1 Introduction

Bladder cancer is the tenth most common type of cancer and ranks thirteenth among the leading causes of cancer-related death. In 2020, there were approximately 573,000 new cases worldwide, with 213,000 deaths attributed to bladder cancer (1). Muscle-invasive bladder cancer (MIBC) accounts for approximately a quarter of all bladder cancer cases, with a median survival of only 5 months without treatment, while patients with high-risk non-muscle-invasive bladder cancer (NMIBC) may have overall survival rates ranging from 28% to 90% depending on the treatment modalities received (2, 3). However, radical cystectomy (RC) combined with pelvic lymph node dissection (PLND) can improve the prognosis of these patients, and is currently the reasonable choice for patients with non-metastatic MIBC and NMIBC with high risk of disease progression (4–6). 

Current guidelines also recommend offering platinum-based adjuvant chemotherapy or adjuvant therapy with Nivolumab for patients with pT3/4 and/or pN+ who have not received neoadjuvant chemotherapy after RC. For patients with pT2 or lower without lymph node metastasis, adjuvant therapy is not recommended (4, 7). However, due to the toxic side effects of platinum-based drugs, the use of adjuvant chemotherapy requires careful consideration of the patient’s renal function, which makes some patients, especially those over 65 years old with insufficient renal function reserve, potentially unsuitable for platinum-based adjuvant chemotherapy (8). Additionally, for patients who experience progression despite RC combined with platinum-based chemotherapy or PD1/PDL1 checkpoint inhibitor therapy, targeted FGFR inhibitors like erdafitinib and antibody-drug conjugates (ADCs) such as Enfortumab vedotin are expected to further improve prognosis (9–11). The response to immunotherapy is closely related to the tumor microenvironment (TME) of bladder cancer, especially in MIBC, a high number of TILs and a low number of cancer-associated fibroblasts (CAFs) can improve the survival rate of patients (12).

Several *in vitro* and *in vivo* studies have shown that Pseudomonas aeruginosa-mannose-sensitive hemagglutinin (PA-MSHA), a genetically modified and inactivated form of Pseudomonas aeruginosa with mannose-sensitive hemagglutinin, can play an anti-tumor role by promoting cancer cell apoptosis, inhibiting cancer cell proliferation, and activating immune cells and cytotoxic effects (13, 14). In addition to its antitumor effects, PA-MSHA has been shown in several studies to treat lymphatic leakage in radical mastectomy (15), gynecologic malignant tumor surgery (16) and radical thyroidectomy (17). Lymphatic leakage is also a common complication after PLND, which may affect the postoperative recovery of patients (18).

Currently, there is a lack of research on the prognosis-related effects of PA-MSHA for patients undergoing RC, as well as a lack of satisfactory treatment for lymphatic leakage following the PLND. Therefore, we conducted a 10-year retrospective cohort study to compare the outcomes and the incidence of postoperative lymphatic leakage between patients who received intraoperative PA-MSHA and those who did not. We hypothesized that spraying PA-MSHA injection in the surgical area before abdominal closure could improve the prognosis of patients and reduce the incidence of lymphatic leakage.

## 2 Patients and method

### 2.1 Study participants

All patients in this study were from two surgical teams in the Urology Department of the Lanzhou University Second Hospital from the years 2013 to 2022. The primary surgeons in bladder cancer radical surgery had at least 15 years of experience, and both teams had the same level of proficiency. This retrospective study was approved by the ethics committee of Lanzhou University Second Hospital and registered at in the Chinese Clinical Trial Registry (ID: ChiCTR2300069891).

### 2.2 Inclusion criteria

(1) Patients undergoing RC and PLND with postoperative pathological staging of T1–4aN0–2M0, along with any type of urinary diversion (Maniz II, neobladder, Bricker, or cutaneous ureterostomy); (2) The pathologic type was urothelial carcinoma; (3) Complete medical records were available.

### 2.3 Exclusion criteria

(1) Neoadjuvant chemotherapy, immuno-target treatment, radiotherapy, or concurrent chemoradiotherapy before surgery; (2) RC after receiving bladder preservation treatment, e.g., conventional transurethral resection of bladder tumor (cTURB) in combination with external beam radiation therapy (EBRT), transurethral resection of bladder tumor (TURBT) or partial cystectomy in combination with chemotherapy or chemoradiotherapy; (3) Perioperative death due to operative complications.

### 2.4 Grouping

(1) PA-MSHA group: 10 ml PA-MSHA (Beijing Wanter Biopharmaceutical Co., Ltd., Beijing, China) was sprayed into the surgical area before abdominal closure, and the drainage tube was opened 3 hours after surgery.
(2) Control group: PA-MSHA was not used during the perioperative period.

### 2.5 Outcome index

#### 2.5.1 Primary outcomes

(1) Overall survival (OS): OS was defined as the interval between the date of operation and death for any reason. If the patient was lost to follow-up, the time to the last follow-up visit was recorded; if the patient was alive during the follow-up, the time interval to the follow-up day was recorded.
(2) Progression-free survival (PFS): PFS was defined as the time from the date of surgery until imaging or clinical evaluation indicated tumor recurrence or metastasis.
(3) Cancer specific survival (CSS): CSS was defined as the interval between the date of the operation and the patient’s death due to bladder cancer. A bladder cancer-related death was defined if patient had evidence of recurrence or metastasis at the last visit, based on visit records and telephone follow-up. If there was no evidence of recurrence within 6 months of the last follow-up, the cause of death was defined as not being related to bladder cancer.

#### 2.5.2 Secondary outcome indicators

(1) Postoperative 3-day drainage volume, pelvic drainage tube retention time, and lymphatic leakage rate: determined by the medical records and nursing records. For patients in whom drainage tubes were removed before discharge, data were obtained from outpatient visit records or telephone follow-up.
(2) Incidence of postoperative adverse events and the rate of visits or re-admissions due to any cause of fever within three months after surgery: from hospital electronic medical records.

### 2.6 Statistical methods

Continuous variables were presented as mean ± standard deviation (SD) for those conforming to a normal distribution, and as median (interquartile range, IQR) for those not conforming. Pearson χ 2 test or Fisher exact test were was used to analyze the classified variables. The effects of different factors on OS, PFS, and CSS were analyzed by the Kaplan-Meier method, and the differences were evaluated using the Log-rank test. Considering potential selection bias, we controlled for differences in baseline characteristics between the two groups by using inverse probability of treatment weighting (IPTW). We used logistic regression models to estimate each patient’s propensity score (PS) for receiving a particular treatment, patients who received PA-MSHA were weighted by 1/PS, while those who did not receive it were weighted by 1/(1−PS). Standardized difference methods and kernel density plots were employed to assess the balance of covariates. Survival outcomes were analyzed using COX regression, and further multivariable COX regression analysis included factors with P <0.2 from the univariate analysis. P < 0.05 was considered statistically significant. R software (version 4.3.3) was used for statistical analysis.

## 3 Results

### 3.1 Patients enrollment and IPTW

**Figure 1:**
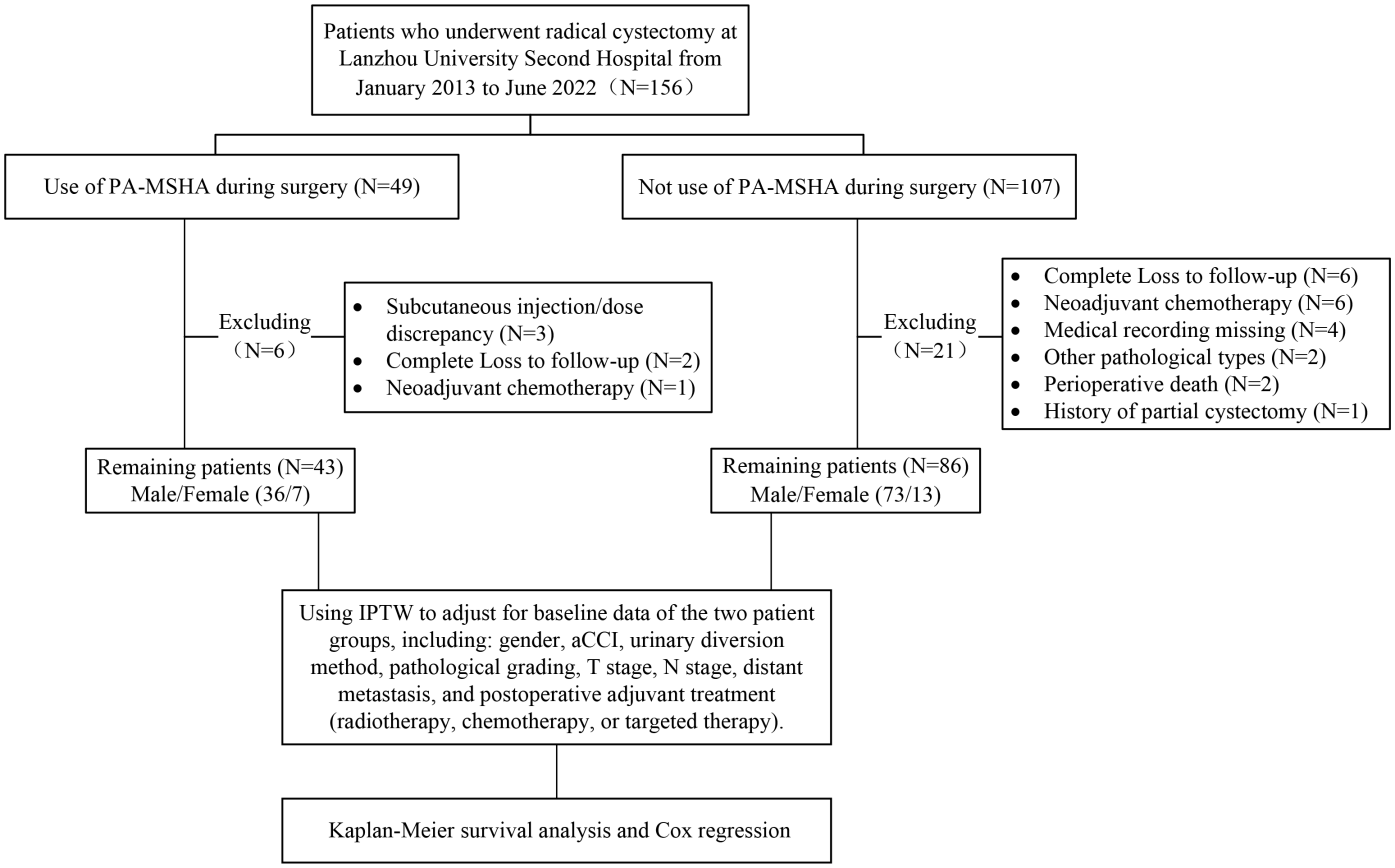
Flow chart of patient enrollment and screening.

We reviewed 156 patients, with 43 cases in the PA-MSHA group and 86 cases in the control group meeting the inclusion criteria (**Figure 1**). In the PA-MSHA group, the average age of patients was 62.1 ± 9.4 years, while in the control group, it was 61.8 ± 8.5 years. Both groups were predominantly male, accounting for 83.72% and 84.88%, respectively. The median aCCI score was 4.0 for both groups. Mainz II procedure was the primary method of urinary diversion, accounting for 83.72% and 61.63%, respectively. Pathological grading and staging primarily consisted of high-grade tumors (86.05% and 88.37% in each group, respectively) and tumors ≤T2 stage (72.09% and 67.45% in each group, respectively). Lymph node metastasis occurred in 13.95% and 20.93% of patients in each group, while distant metastasis occurred in 25.58% and 31.40% of patients, respectively. In the PA-MSHA group, 5 patients received postoperative adjuvant treatment, including 2 patients receiving postoperative chemotherapy and 3 patients receiving postoperative chemotherapy combined with bevacizumab. In the control group, 15 patients received adjuvant treatment, including 14 patients receiving postoperative chemotherapy and 1 patient receiving chemotherapy combined with vedolizumab. We further used IPTW to balance baseline differences between the two groups, resulting in all variables having a standardized mean difference (SMD) of less than 0.1 after IPTW adjustment (**Table 1**; **Figure 2**).

**Table 1:** Clinical baseline characteristics of the PA-MSHA group and Control group.

	All data				After IPTW			
	Control (n=86)	PA-MSHA (n=43)	*P*	SMD	Control (n=86)	PA-MSHA (n=43.02)	*P*	SMD
** Male (%)**	73 (84.88)	36 (83.72)	1	0.032	72.88 (84.75)	36.87 (85.71)	0.884	0.027
** Female(%)**	13 (15.12)	7 (16.28)			13.12 (15.25)	6.15 (14.29)		
**aCCI (median [IQR])**	4.00 [3.00, 4.00]	4.00 [3.00, 4.50]	0.831	0.083	4.00 [3.00, 4.00]	4.00 [3.00, 5.00]	0.855	<0.001
**Urinary diversion (%)**			0.057	0.572			0.169	0.493
** Bricker**	11 (12.79)	3 (6.98)			12.57 (14.61)	2.84 (6.59)		
** Mainz II**	53 (61.63)	36 (83.72)			54.10 (62.90)	34.11 (79.28)		
** Ileal neobladder**	6 (6.98)	0 (0.00)			5.49 (6.38)	0.00 (0.00)		
** Cutaneous ureterostomy**	16 (18.60)	4 (9.30)			13.84 (16.10)	6.08 (14.12)		
**High grade (%)**	76 (88.37)	37 (86.05)	0.925	0.07	75.56 (87.87)	38.13 (88.62)	0.896	0.024
**Low grade (%)**	10 (11.63)	6 (13.95)			10.44 (12.13)	4.89 (11.38)		
**pT stage (%)**			0.859	0.166			0.89	0.153
** T1**	20 (23.26)	9 (20.93)			20.26 (23.56)	8.22 (19.10)		
** T2**	38 (44.19)	22 (51.16)			38.72 (45.03)	22.27 (51.77)		
** T3**	19 (22.09)	9 (20.93)			18.53 (21.54)	9.15 (21.27)		
** T4**	9 (10.47)	3 (6.98)			8.48 (9.87)	3.38 (7.85)		
**pN+ (%)**	18 (20.93)	6 (13.95)	0.472	0.185	16.22 (18.87)	8.64 (20.09)	0.881	0.031
**Metastasis = Yes (%)**	27 (31.40)	11 (25.58)	0.633	0.129	25.53 (29.69)	13.15 (30.57)	0.923	0.019
**Adjuvant therapy^#^= Yes (%)**	15 (17.44)	5 (11.63)	0.547	0.166	13.38 (15.56)	6.65 (15.45)	0.988	0.003

#Adjuvant therapy includes radiotherapy, chemotherapy, and targeted therapy.

**Figure 2:**
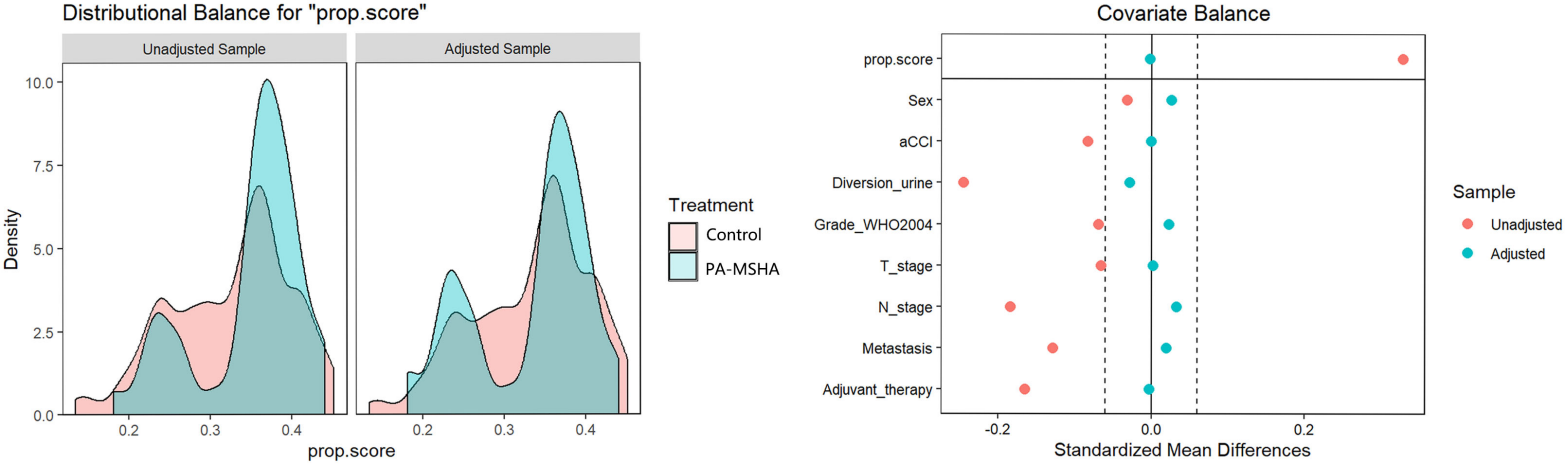
Inverse probability treatment weighting (IPTW) adjustment for the impact on the preoperative clinical characteristic distribution of patients undergoing radical cystectomy, whether receiving PA-MSHA treatment or not, with the left side showing Kernel density and the right side showing the love plot of SMD; after adjustment, the SMD between the two groups is less than 0.1.

### 3.2 PA-MSHA may improve OS, PFS and CSS in patients undergoing radical cystectomy for bladder cancer

Of the 43 people who completed follow-up in the PA-MSHA group, there were 17 deaths, including 15 from bladder cancer, 1 from cardiovascular disease, and 1 from cerebral hemorrhage. Of the 86 patients in the control group, 45 died, including 41 from bladder cancer, 3 from renal failure, 1 from gastric cancer. The median follow-up time was 54 months in the PA-MSHA group and 48 months in the control group.

**Figure 3:**
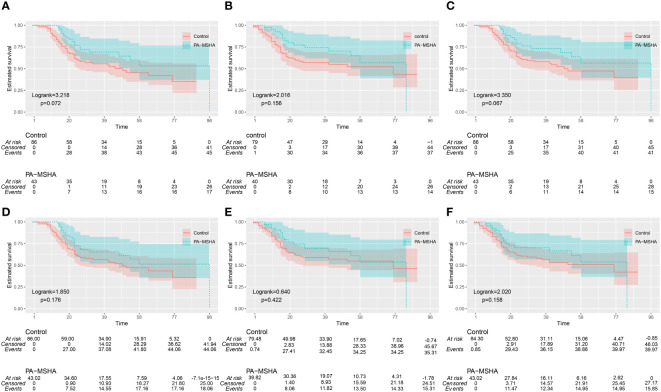
Kaplan-Meier survival curves for the PA-MSHA group and the Control group. **(A-C)** OS (Overall Survival), PFS (Progression-Free Survival), and CSS (Cancer-Specific Survival) before IPTW adjustment; **(D-F)** OS, PFS, and CSS after IPTW adjustment.

Since patients in the PA-MSHA group did not reach the median survival time, we report the 25th and 75th percentile survival times as 96 months and 26 months, respectively, with OS rates of 69.1% at 3 years and 53.2% at 5 years. The control group had a median survival time of 49 months (95% CI: 23.3–74.8), with OS rates of 55.6% at 3 years and 45.3% at 5 years. Kaplan-Meier survival analysis shows that the PA-MSHA group appears to have better median OS, 3-year, and 5-year OS rates compared to the control group, although the difference is not statistically significant (Log-rank=3.218, P=0.072) (**Figure 3A**). In the PA-MSHA group and the control group, 3 and 5 individuals, respectively, did not undergo scheduled outpatient follow-up visits. At the last follow-up, multiple systemic metastases were already present, so the exact progression time was unclear for these 8 patients, who were therefore excluded from the analysis. Among the remaining patients, the three-year and five-year PFS rates for the PA-MSHA group were 74.4% and 56.8%, respectively, while for the control group, the three-year and five-year PFS rates were 57.1% and 52.2%, respectively. There was no statistically significant difference between the two groups (Log-rank=2.016, P=0.156) (**Figure 3B**). In the PA-MSHA group, the 3-year and 5-year CSS rates were 73.3% and 56.5%, respectively, compared to 58.0% and 47.3% in the control group, showing a potential advantage but with no statistically significant difference (P=0.067, Log-rank=3.350) (**Figure 3C**).

After IPTW adjustment, the 3-year and 5-year OS rates for the PA-MSHA group were 65.5% and 51.3%, respectively, while for the control group they were 56.6% and 47.2%. There was no statistically significant difference between the two groups (Log-rank = 1.850, P=0.176) (**Figure 3D**). After IPTW adjustment, the 3-year and 5-year PFS rates for the PA-MSHA group were 69.4% and53.7%, respectively, while for the control group they were 59.1% and 54.9%. There was no statistically significant difference between the two groups (Log-rank=0.640, P=0.422) (**Figure 3E**). After IPTW adjustment, the 3-year and 5-year CSS rates for the PA-MSHA group were 69.9% and 54.8%, respectively, while for the control group they were 59.0% and 49.2%. There was no statistically significant difference between the two groups (Log-rank = 2.020, P=0.158) (**Figure 3F**).

**Figure 4:**
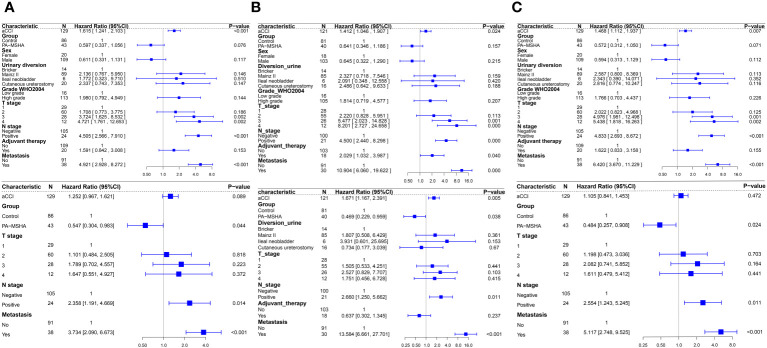
Forest plots of Cox regression analysis affecting patient OS, PFS, and CSS, with univariate analysis above and multivariate analysis below. **(A)** Univariate and multivariate Cox regression analysis for OS; **(B)** Univariate and multivariate Cox regression analysis for PFS; **(C)** Univariate and multivariate Cox regression analysis for CSS.

Univariate Cox regression analysis indicated that higher aCCI (HR:1.615, 95%CI: 1.241–2.103, P<0.001), tumors in T3 stage or above (When staged as T3, HR: 3.724, 95% CI: 1.625–8.532, P=0.002; when staged as T4, HR: 4.721, 95% CI: 1.761–12.653, P=0.002), presence of lymph node metastasis (HR: 4.505, 95%CI:2.566–7.910, P<0.001), and distant metastasis (HR: 4.921, 95%CI:2.928–8.272, P<0.001) are factors of poor OS for patients. The use of PA-MSHA appears to improve patients’ OS (HR: 0.597, 95%CI: 0.337–1.056, P=0.076) and CSS (HR: 0.572, 95%CI: 0.312–1.050, P=0.071) but does not improve patients’ PFS (HR: 0.641, 95%CI: 0.346–1.186, P=0.157). Multivariate Cox regression analysis indicates that lymph node metastasis and distant metastasis are poor prognostic factors for patients, while the use of PA-MSHA can improve patients’ OS (HR: 0.547, 95%CI: 0.304–0.983, P=0.044), PFS (HR: 0.469, 95%CI: 0.229–0.959, P=0.038) and CSS (HR: 0.484, 95%CI: 0.257–0.908, P=0.024) (**Figures 4A–C**).

### 3.3 PA-MSHA group had a shorter pelvic drainage tube retention and not increase infection rates

The postoperative fever rates in the PA-MSHA group and the control group were 53.5% (23/43) and 55.8% (48/86) respectively, with no statistically significant difference between the two groups (P=0.802). Among patients in the PA-MSHA group, two patients developed unexplained rashes, while no such events occurred in the control group. There was no significant difference in postoperative abdominal pain, abdominal distension and nausea between the two groups (P=0.350). The postoperative lymphatic leakage rates in the PA-MSHA group and the control group were 18.6% (8/35) and 15.1% (84.9%), respectively, with no statistically significant difference (P=0.613). The postoperative pelvic drainage volumes at 3 days were 470 (440) ml for the PA-MSHA group and 462.5 (430) ml for the control group, with no statistical difference (P=0.814). However, the median drainage tube retention time in the PA-MSHA group (7.0 days) was shorter compared to the control group (median time 9.0 days) (P=0.021). Lastly, the rehospitalization or re-visit rates within 90 days postoperatively due to any fever-related reasons were 20.9% (9/43) for the PA-MSHA group and 19.8% (17/86) for the control group, with no statistically significant difference between the groups (P=0.877) (**Table 2**).

**Table 2:** Postoperative events occurrence in the PA-MSHA group and Control group.

Characteristics	PA-MSHA	Control	*P*
Lymphatic leakage (Yes/No)	8 (18.6%)/35 (81.4%)	13 (15.1%)/73 (84.9%)	0.613
Pelvic drainage volume 3 days after operation (ml)	470 (440)	462.5 (430)	0.814
Drainage tube retention time (days)	7.0 (7.0)	9.0 (7.0)	0.021
Postoperative fever events (Yes/No)	23 (53.5%)/20 (46.5%)	48 (55.8%)/38 (44.2%)	0.802
Re-admission or visit due to fever within 90 days postoperatively (n%)	9 (20.9%)/34(79.1%)	17 (19.8%)/69 (80.2%)	0.877	

## 4 Discussion

In this study, we evaluated the impact of PA-MSHA on the OS, PFS, and CSS of patients undergoing radical cystectomy for bladder cancer. Our analyses, employing Kaplan-Meier survival analysis and Cox univariate analysis, revealed that patients treated with PA-MSHA exhibited prolonged OS and CSS compared to the control group, although the differences did not reach statistical significance. However, upon conducting multivariable Cox regression analysis, these differences became significant. Furthermore, for PFS, the use of PA-MSHA was significantly associated with extended PFS in the multivariable Cox regression analysis. Given the marginal p-values associated with these positive outcomes and the relatively small sample size in our cohort, despite the strong comparability demonstrated by baseline characteristics between the two groups, we contemplated employing IPTW to enhance the robustness of our results and reduce estimation bias. However, following IPTW adjustment, the observed trend of diminished differences between the PA-MSHA group and the control group in OS, PFS, and CSS was noteworthy. On one hand, this observation may indicate that, under the premise of IPTW adjustment based on the selected parameters in this study, a more balanced baseline might render prognostic factor disparities statistically insignificant. On the other hand, the lack of statistical significance in the differences could be attributed to the inadequacy of the sample size and insufficient statistical power. Nevertheless, both in the original cohort and the cohort post-IPTW adjustment, patients in the PA-MSHA group demonstrated a trend toward better prognosis. Currently, there are few studies on the prognosis of tumors with PA-MSHA. A recent study showed that adding PA-MSHA to neoadjuvant chemotherapy can improve the objective response rate (ORR) of HER2-negative breast cancer, but there was no significant difference in pathological complete response (pCR) and survival outcomes. In addition, the use of PA-MSHA can increase serum interferon-γ levels and the percentage of peripheral blood T cells, CD8+/CD4+T cells, CD8+CD28+T cells, and natural killer (NK) cells, and decrease serum interleukin 4 levels, suggesting that PA-MSHA may exert its anti-tumor effects through immune modulation (19). In an earlier study on the treatment of advanced non-small cell lung cancer (NSCLC), the combination of PA-MSHA with chemotherapy improved the objective response rate (ORR) without affecting the 1-year survival rate, and did not cause severe adverse events or impact patient quality of life (20). Recent studies have shown that PA-MSHA can regulate immune response by enhancing T cell response, promoting dendritic cell maturation and M1 macrophage polarization, promoting apoptosis of bladder tumor cells, inhibiting their proliferation, invasion, and migration, and possibly by inducing inflammatory responses in the tumor microenvironment and increasing sensitivity to tumor PD-1 therapy (21–23). However, we were unable to obtain information on blood tests at the same time points after surgery or in the outpatient review, so we could not assess the changes in the proportion of immune cells in the blood after PA-MSHA treatment, which requires future research.

Previous studies reported that fever, pain and rash were the most common adverse reactions after the use of PA-MSHA (16, 24). However, in our study, we found that there was also no difference between the two groups in the incidence of postoperative fever and the rate of readmission due to fever within 3 months after surgery. In addition, there was no significant difference in gastrointestinal adverse reactions such as abdominal pain, abdominal distension and nausea between the two groups. Due to incomplete information in some medical records, we could not further obtain more detailed information such as the cause, degree, and duration of fever. In addition, the postoperative fever and pain may mask the adverse reactions of the drug itself.

The incidence of lymphatic leakage in different urological procedures ranges from 0.77% to 15% (25).Two retrospective studies have found that the incidence of lymphatic leakage after RC was 22.2% for standard PLND (26) and 39.5% for enlarged PLND (27). Long-term lymphatic leakage can lead to malnutrition, disturbance of water and electrolyte, immune impairment, prolonged hospitalization and even death (28). Currently, conservative treatment is mainly used for postoperative lymphatic leakage, including a low-fat diet, parenteral nutrition, drug therapy such as somatostatin or octreotide, and radiation interventional therapy or surgical treatment are also optional (29). Previous studies have found that spraying 2ml of undiluted PA-MSHA on postoperative wounds of thyroid or breast cancer while clamping the drainage tube for at least half an hour can effectively prevent and treat postoperative lymphatic leakage without other serious adverse reactions except for drug control of fever and pain (17, 24, 30). However, in our study, spraying 10 ml undiluted PA-MSHA on the surgical wound did not reduce the incidence of lymphatic leakage and pelvic drainage volume at 3 days after RC, but it shortened drainage tube retention time. We need to take these results seriously. On one hand, unlike radical mastectomy or radical thyroid cancer, radical bladder surgery is more invasive. The occluded capillaries and lymphatic vessels on the extensive peritoneal wound may exude again due to the absence of pneumoperitoneum, the loss of electrocoagulation escus, or tissue necrosis. In fact, some patients may still experience a sudden increase in drainage volume 3 days after surgery, so only comparing the amount of drainage fluid within 3 days after surgery may not represent the total drainage fluid. However, we could not obtain additional information because the electronic medical record did not record drainage volumes beyond day 3. Nevertheless, the utilization of PA-MSHA can decrease the retention time of the pelvic drainage tube, potentially indicating a reduction in pelvic drainage volume. Additionally, due to the anticipated preventative impact of PA-MSHA, it may proactively deter the onset of lymphatic leakage, a fact that warrants further investigation. On the other hand, the dosage, retention time, and use timing of our PA-MSHA were inconsistent with other studies. The efficacy of PA-MSHA and whether there is a dose-time-dependent relationship between PA-MSHA and adverse reactions deserve further investigation.

Our study has the following limitations. First, the sample size of this study was relatively small and the statistical power was limited. Therefore, prospective studies with larger sample sizes are needed for verification. Second, immune-related biomarkers such as serum cytokines and immune cell subsets were not evaluated in this study. Finally, recall bias could not be completely eliminated due to the retrospective design of this study.

## 5 Conclusion

In conclusion, our team used PA-MSHA as an adjuvant therapy in patients undergoing radical cystectomy and found that PA-MSHA is safe and may improve OS, PFS, and CSS in patients undergoing radical cystectomy. Moreover, PA-MSHA did not reduce the drainage volume of patients within 3 days after surgery or the incidence of lymphatic leakage, but it can shorten the retention of pelvic drainage tube.

## Data availability statement

The raw data supporting the conclusions of this article will be made available by the authors, without undue reservation.

## Ethics statement

The studies involving humans were approved by Lanzhou University Second Hospital. The studies were conducted in accordance with the local legislation and institutional requirements. Written informed consent for participation was not required from the participants or the participants’ legal guardians/next of kin because this study is a retrospective cohort study and waiver of informed consent has been applied for.

## Author contributions

XZ: Writing – review & editing, Writing – original draft, Software, Project administration, Methodology, Formal analysis, Conceptualization. ZP: Writing – review & editing, Methodology, Formal analysis, Data curation, Conceptualization. JR: Writing – review & editing, Formal analysis, Data curation. JS: Writing – review & editing, Formal analysis, Data curation. WL: Writing – review & editing, Data curation. YS: Writing – review & editing, Data curation. WM: Writing – review & editing, Data curation. LZ: Writing – review & editing, Data curation. HD: Writing – review & editing, Resources. YZ: Writing – review & editing, Resources. JT: Writing – review & editing, Resources. ZW: Writing – review & editing, Supervision, Software, Resources, Project administration, Methodology, Funding acquisition, Conceptualization.
